# Comparison of radiologic findings between SARS-CoV-2 and other respiratory tract viruses in critically ill children during the COVID-19 pandemic

**DOI:** 10.55730/1300-0144.5818

**Published:** 2024-03-11

**Authors:** Oktay PERK, Tanıl KENDİRLİ, Emel UYAR, Birsel ŞEN AKOVA, Hatice ALBAYRAK, Hasan AĞIN, Ebru Atike ONGUN, Eşe Eda TURANLI, Sare Güntülü ŞIK, Şahin SİNCAR, Gürkan BOZAN, Demet DEMİRKOL, Nazan ÜLGEN TEKEREK, Mey TALİP, Arzu OTO, Feyza İNCEKÖY GİRGİN, Ferhat SARI, Nurettin Onur KUTLU, Altan GÜNEŞ, Ömer Suat FİTÖZ, the PI-COVID-19 Study Group

**Affiliations:** 1 Department of Pediatric Intensive Care, Ankara City Hospital, Ankara, Turkiye; 2 Department of Pediatric Intensive Care, Ankara University School of Medicine, Ankara, Turkiye; 3 Department of Pediatric Radiology, Ankara University School of Medicine, Ankara, Turkiye; 4 Department of Pediatric Intensive Care, Ondokuz Mayıs University School of Medicine, Samsun, Turkiye; 5 Department of Pediatric Intensive Care, Dr. Behçet Uz Health Training and Research Hospital, İzmir, Turkiye; 6 Department of Pediatric Intensive Care, Antalya Training and Research Hospital, Antalya, Turkiye; 7 Department of Pediatric Intensive Care, Ege University School of Medicine, İzmir, Turkiye; 8 Department of Pediatric Intensive Care, Acıbadem Mehmet Ali Aydınlar University School of Medicine, İstanbul, Turkiye; 9 Department of Pediatric Intensive Care, Elazığ Fethi Sekin City Hospital, Elazığ, Turkiye; 10 Department of Pediatric Intensive Care, Eskişehir Osmangazi University School of Medicine, Eskişehir, Turkiye; 11 Department of Pediatric Intensive Care, İstanbul University School of Medicine, İstanbul, Turkiye; 12 Department of Pediatric Intensive Care, Akdeniz University School of Medicine, Antalya, Turkiye; 13 Department of Pediatric Intensive Care, Prof. Dr Cemil Taşcıoğlu City Hospital, İstanbul, Turkiye; 14 Department of Pediatric Intensive Care, The University of Health Sciences Bursa Yüksek Ihtisas Training and Research Hospital, Bursa, Turkiye; 15 Department of Pediatric Intensive Care, Marmara University School of Medicine, İstanbul, Turkiye; 16 Department of Pediatric Intensive Care, Mustafa Kemal University Tayfur Ata Sökmen School of Medicine, Hatay, Turkiye; 17 Department of Pediatric Intensive Care, İstanbul Başakşehir Çam ve Sakura City Hospital, İstanbul, Turkiye; 18 Department of Pediatric Radiology, Ankara City Hospital, Ankara, Turkiye

**Keywords:** SARS-CoV-2, COVID-19, respiratory system, tomography, pediatric intensive care units

## Abstract

**Background/aim:**

This study was planned because the radiological distinction of COVID-19 and respiratory viral panel (RVP)-positive cases is necessary to prioritize intensive care needs and ensure non-COVID-19 cases are not overlooked. With that purpose, the objective of this study was to compare radiologic findings between SARS-CoV-2 and other respiratory airway viruses in critically ill children with suspected COVID-19 disease.

**Materials and methods:**

This study was conducted as a multicenter, retrospective, observational, and cohort study in 24 pediatric intensive care units between March 1 and May 31, 2020. SARS-CoV-2- or RVP polymerase chain reaction (PCR)-positive patients’ chest X-ray and thoracic computed tomography (CT) findings were evaluated blindly by pediatric radiologists.

**Results:**

We enrolled 225 patients in the study, 81 of whom tested positive for Coronovirus disease-19 (COVID-19) caused by severe acute respiratory syndrome coronavirus-2 (SARS-CoV-2). The median age of all patients was 24 (7–96) months, while it was 96 (17–156) months for COVID-19-positive patients and 17 (6–48) months for positive for other RVP factor (p < 0.001). Chest X-rays were more frequently evaluated as normal in patients with SARS-CoV-2 positive results (p = 0.020). Unilateral segmental or lobar consolidation was observed more frequently on chest X-rays in rhinovirus cases than in other groups (p = 0.038). CT imaging findings of bilateral peribronchial thickening and/or peribronchial opacity were more frequently observed in RVP-positive patients (p = 0.046).

**Conclusion:**

Chest X-ray and CT findings in COVID-19 patients are not specific and can be seen in other respiratory virus infections.

## 1. Introduction

One of the world’s biggest challenges during the Coronavirus Disease 2019 (COVID-19) pandemic, caused by severe acute respiratory syndrome coronavirus-2 (SARS-CoV-2), is how to diagnose the disease in suspected patients and determine who needs testing. Currently, the test used for detecting SARS-CoV-2 is the polymerase chain reaction (PCR) test conducted on a nasopharyngeal swab. The effectiveness of the tests depends on the accuracy of the test and how the test results will affect the treatment. Although reverse transcription polymerase chain reaction (RT-PCR) is a highly sensitive and specific test, it requires highly skilled personnel and special instruments. Otherwise, the test samples may become contaminated, leading to inaccurate or unreliable results. In addition, the sensitivity of the test may have variations depending on the duration from the onset of disease symptoms and the severity of the disease. Therefore, the sensitivity of the test may change from 60% to 95% [1,2,3].

Chest X-rays are generally not preferred to CT scans because of their low sensitivity in detection of pulmonary infiltration. Initially, findings on chest CT were accepted as diagnostic in patients with or without respiratory distress, with a history of exposure to the virus, or in patients with other symptoms of COVID19. At beginning of COVID-19 pandemic, thorax CT was the sole method used, but it later became apparent that it was not the only method available and was not sufficient for a definitive diagnosis in adults of all ages. Additionally, its use for COVID-19 diagnosis in children remains controversial. Thoracic CT has not been routinely performed in pediatric patients of suspected COVID-19. Apart from SARS-CoV-2 (COVID-19, caused by severe acute respiratory syndrome cornavirus-2), there are many other viruses that can cause pneumonia, and even death. These viruses can also lead to epidemics and pediatric acute respiratory distress syndrome (PARDS). Some examples are influenza virus, respiratory syncytial virus (RSV), rhinovirus, adenovirus, parainfluenza virus, metapneumovirus, bocavirus. However, unlike COVID-19, thoracic CT may not have diagnostic advantages in diseases caused by these viruses [4,5].

Reported observations have shown that viruses other than SARS-CoV-2 may also create ground-glass opacities (GGOs), multiple patchy consolidations, and peripheral or central involvement in the lung parenchyma similar to COVID-19 [6].

In this multicenter study, we investigated the diagnostic sensitivity and specificity of chest X-rays and thoracic CT imaging for the differential diagnosis and clinical significance in pediatric intensive care patients with suspected SARS-CoV-2 or respiratory tract virus infections during the COVID-19 outbreak.

## 2. Materials and methods

This study was designed as a retrospective multicenter cohort study. Twenty-four pediatric intensive care units (PICUs) across Türkiye participated in this study. Patients between the ages of 1 month and 18 years of age who were hospitalized in PICUs between March 1 and May 31, 2020 with respiratory system symptoms and positive real-time reverse transcriptase PCR (RT-PCR) tests for either SARS-CoV-2 or respiratory viral panel (RVP) viruses [including RSV, rhinovirus, influenza virus (including H1N1), adenovirus, and metapneumovirus, among others] were included in the study. Patients with inconclusive SARS-CoV-2 PCR tests or those with positive thoracic CT findings for SARS-CoV-2 but negative PCR tests were also excluded from the study. Patients who had positive COVID-19 serology but negative PCR tests were also excluded from the study. Patients with positive serologic findings for both COVID-19 and RVP PCR at the same time were also excluded from the study.

**Figure 1:**
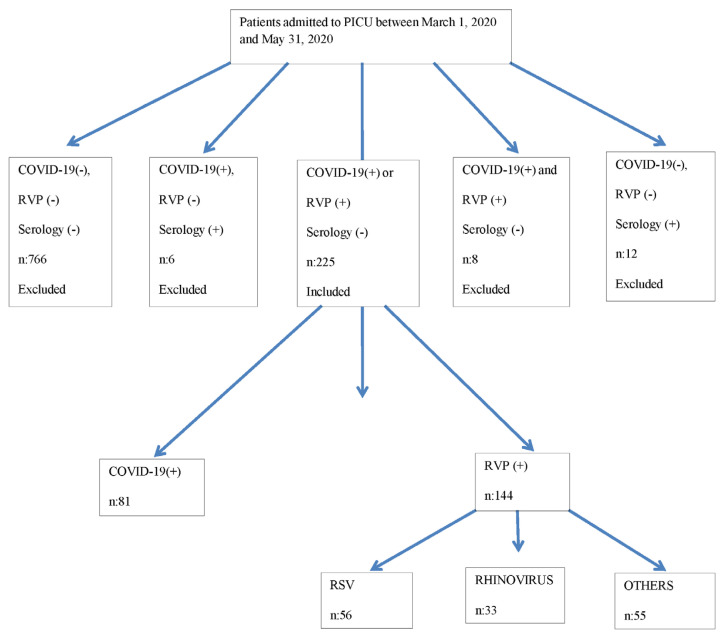
The diagram is as follows. The study flow chart illustrating the included or excluded patients. RVP: Respiratory viral panel, RSV: respiratory syncytial virus.

Patients were divided into two groups: SARS-CoV-2-positive (Group 1) and RVP-positive (Group 2). The method targeting the RNA-dependent RNA polymerase (RdRp) gene using the Bio-Speedy COVID-19 RTqPCR Detection Kit (Bioeksen, İstanbul, Türkiye) for SARS-CoV-2 and RVP [upper RVP was studied with five-tube multiplex for detection of influenza A virus; influenza A (H1N1) virus (swine-lineage); influenza B virus; human rhinovirus; human coronaviruses NL63, 229E, OC43, and HKU1; human parainfluenza viruses 1, 2, 3, and 4; human metapneumovirus A/B; human bocavirus; human RSV A/B; human adenovirus; enterovirus; human parechovirus; and Mycoplasma pneumonia] and internal control using Fast track resp 21; Multiplex real-time PCR for detection of pathogen genes by TaqMan technology (Rotor-gene, California, USA) were used to analyze the patients’ nasopharyngeal swab samples. If at least one of these tests was positive, it was accepted as significant. The flow chart of the study is presented in Figure 1.

Pediatric risk of mortality score III (PRISM III), pediatric logistic organ dysfunction score2 (PELOD-2), and pediatric multiple organ dysfunction score (P-MODS) can be used to dynamically assess pediatric patients and accurately determine the risk of death or potentially serious complications in critically ill patients of all age groups, including pediatric patients. Calculated with Oxygen Saturation Index (OSI) ([FiO2 × Mean airway pressure × 100]/SpO_2_) [7]. PRISM-III, PELOD-2, P-MODS, and OSI scores were calculated in accordance with the literature.

### 2.1. Radiological evaluation

In both groups, X-rays and CT scans that were performed within 48 h of admission to the PICU were evaluated by two radiologists independently (A.G. and B.S.A., with eight and two years of experience in pediatric radiology, respectively) for both diagnostic purposes. The final decision was made by a senior pediatric radiologist for inconclusive results (S.F. with 22 years of experience in pediatric radiology). The radiologists were blinded to the diagnosis or PCR results of the patients.

The radiologists filled out a standard form of radiological for each patient. The form was inspired by an example of the reporting chart recommended by a group of international pediatric thoracic radiologists under the joint consensus “International Expert Consensus Statement on Chest Imaging in Pediatric COVID-19 Patient Management: Imaging Findings and Imaging Study Reporting, and Imaging Study [Recommendations] [8]. Chest X-ray findings were classified as typical (bilateral distribution peripheral and/or subpleural GGOs and/or consolidation), indeterminate (unilateral peripheral or peripherocentral GGOs and/or consolidation; bilateral peribronchial thickening and/or peribronchial opacities; multifocal or diffuse GGOs and/or consolidation without specific distribution), and atypical (unilateral segmental or lobar consolidation, central unilateral or bilateral GGOs and/or consolidation, single round consolidation, pleural effusion and lymphadenopathy) according to guideline. Typical (bilateral peripheral subpleural ground-glass infiltrates and/or consolidation and/or halo sign) and indeterminate findings (multifocal or diffuse ground-glass infiltration and/or consolidation, unilateral peripheral or peripheral central ground-glass infiltrations and/or consolidation and/or crazy paving pattern) for CT were evaluated according to these criteria. Thoracic ultrasonography and magnetic resonance imaging examination results for investigation of those patients were not included in the study.

This work was authorized by the Ministry of Health (Ethics Committee-20-568), and the approval of the local ethics committee (Ethics Committee no: 568) was obtained.

### 2.2. Statistical analysis and method

First, descriptive parameters (mean, median, number, and percentage) of the variables were evaluated. The numeric variables were checked to determine whether they fit the normal distribution. While comparing the two groups, Student’s t-test was used for numerical variables with a normal distribution. The Mann–Whitney U test was used for numerical variables that were not normally distributed. The chi-square test was performed to compare categorical variables. A p-value <0.05 was considered statistically significant. SPSS v. 17.0 for Windows. (IBM Corp., Armonk, NY; USA) was used for statistical analysis.

## 3. Results

**Figure 2:**
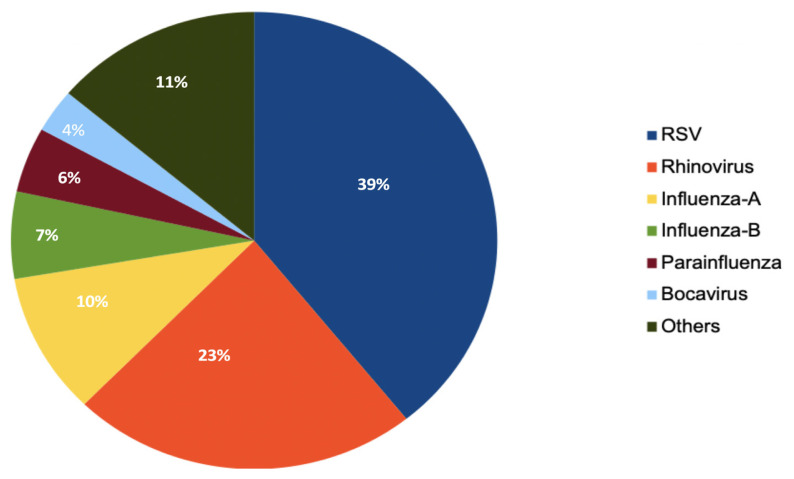
Respiratory viral panel. Diagram illustrating the viral etiologies detected in patients. RSV: 56, Rhinovirus: 33, Influenza-A: 15, Influenza-B: 10, Parainfluenza: 9, Bocavirus: 6, Others: 15.

A total of 225 pediatric patients who were PCR-positive for COVID-19 or RVP were included in the study. Eighty one patients were positive for COVID-19 and 144 patients were positive for one of the other viral infectious agents studied in RVP. Among respiratory tract viruses, the most common cause was RSV detected in 56 patients, and the second most common agent was rhinovirus, which was positive in 33 patients. Figure 2 presents a pie chart showing the distribution of RVP agents detected in our study. The median age of all patients was 24 (7–96) months, while it was 96 (17–156) months for COVID-19-positive patients and 17 (6–48) months for patients positive for other respiratory viral panel (RVP) factors (p < 0.001). One-hundred-thirty (57.8%) of all patients, 44 (54.3%) were positive for COVID-19, and 86 (59.7%) of the patients with positive RVP factor were male (p < 0.431). Thirty-four (42.0%) of the COVID-19-positive patients had a history of contact with COVID-19 patients. One-hundred-thirty-one (59%) patients, including 46 (58.2%) COVID-19-positive patients and 85 (59.4%) RVP-positive patients, had additional diseases (p = 0.86). PRISM and PELOD scores were statistically significantly higher in COVID-19-positive patients than in RVP-positive patients. The median OSI was 6 (3.6–12) in all patients, with a statistically significant difference determined compared to RVP-positive patients (7.75, range 5–13), and COVID-19-positive patients (3.65, range 0–8.35) (p = 0.016). The most common symptoms reported by both groups were shortness of breath (76%), fever (47.1%), and cough (40%). Shortness of breath and fever symptoms were statistically significantly higher in patients with RVP compared to COVID-19-positive patients. Age, sex, history of contact with a contaminated person, presence of other diseases, PRISM PELOD and OSI scores at presentation, and the most common symptoms at presentation are detailed in Table 1.

### 3.1. Chest X-rays of the patients (COVID-19/RVP)

**Figure 3:**
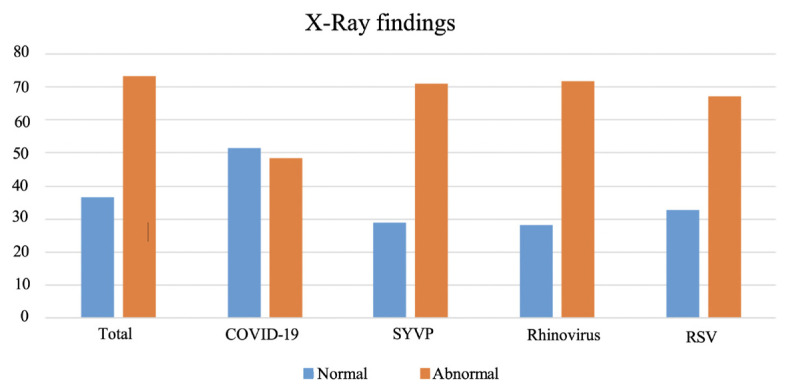
Findings of normal and abnormal chest X-ray ratios in patients infected with COVID-19, other viruses, and the total patient population.

**Figure 4:**
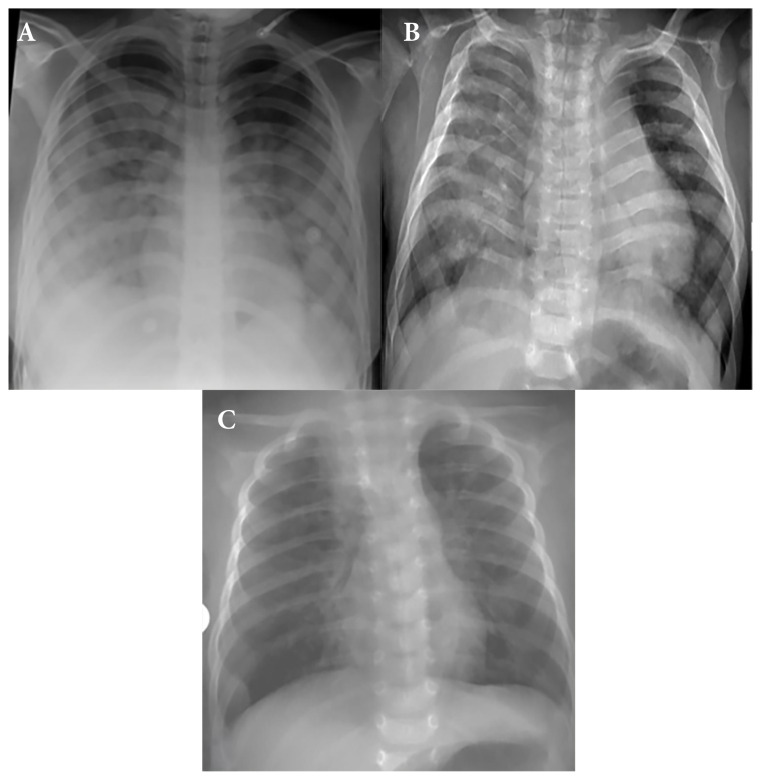
**A)** Patient 221: 14-year-old girl with autoimmune polyglandular syndrome. Bilateral mainly peripheral ground glass opacities was seen at middle and lower zones, on chest X-ray, diagnosed as COVID-19, **B)** Patient 40: 1-year-old boy with Schinzel Giedion syndrome. Multifocal infiltrations were seen on X-ray, predominantly on the right side. Hyperinflation was prominent especially in the left lung. Rhinovirus was detected at RVP tests, **C)** Patient 171: 1-month-old immigrant boy who had a history of contact with COVID-19 positive patient. Chest X-ray showed bilateral peribronchial opacities and overinflation. Diagnosis of RSV pneumonia was made after RVP tests.

A total of 213 patients had chest X-ray exam. Among them, the chest X-rays of 78 (36.6%) patients—38 (51.4%) with COVID-19 positive and 40 (28.8%) with RVP positive results—showed findings within the normal range (p = 0.020) (Figure 3). Bilateral peribronchial thickening or pulmonary opacities were detected in 30 (21.6%) of the RVP-positive patients and seven (9.5%) of the COVID-19-positive cases, with a statistically significant difference (p = 0.042). Multifocal or diffuse ground glass infiltration and/or consolidation [46 (21.6%)] and unilateral peripheral or peripheral central ground glass infiltration and/or consolidation [27 (12.7%)] were the most common findings on chest radiographs without statistically significant differences between the groups (p = 0.223, p = 0.359, respectively) (Figure 4). There was no statistically significant difference in the other radiological parameters, such as bilateral peripheral subpleural ground-glass infiltrates and/or consolidation, unilateral segmental or lobar consolidation, central unilateral or bilateral ground-glass infiltration and/or consolidation, pleural effusion, and lymphadenopathy (p = 0.520, p = 0.563, p = 0.589, p = 1000, p = 1000, respectively). Chest X-ray findings and statistical comparisons of the patients according to the groups are presented in Table 2.

Chest X-rays in COVID-19-positive patients had more normal findings compared to rhinovirus and RSV-positive patients, and this difference was statistically significant (p = 0.029) (Figure 3). Unilateral segmental or lobar consolidation was more common in rhinovirus cases [six (18.8%)], and the difference was statistically significant (p = 0.038). Bilateral peribronchial thickening and/or peribronchial opacities, multifocal or diffuse ground-glass infiltrations and/or consolidation, unilateral peripheral or peripheral and central ground-glass infiltration and/or consolidation, and central unilateral or bilateral ground-glass infiltration and/or consolidation were common in all groups. There were no statistically significant differences for these chest X-ray features (p = 0.054, p = 0.793, p = 0.335, p = 0.230, respectively). Chest X-ray findings and statistical comparisons of the patients according to the groups are detailed in Table 2.

### 3.2. Computed tomography of the patients (COVID-19/RVP)

**Figure 5:**
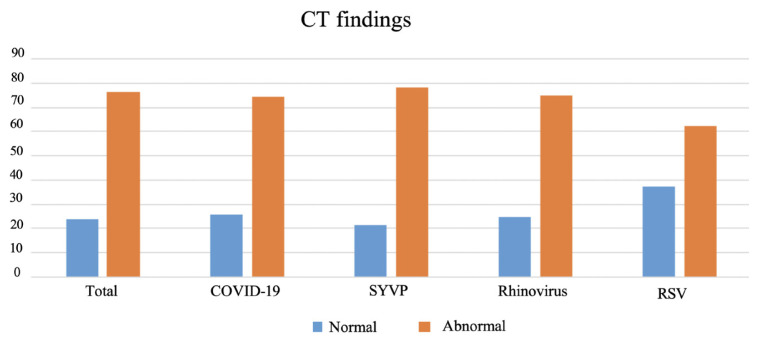
Normal and abnormal thorax computed tomography ratios in COVID-19 and other viruses infected patients and total patients

**Figure 6:**
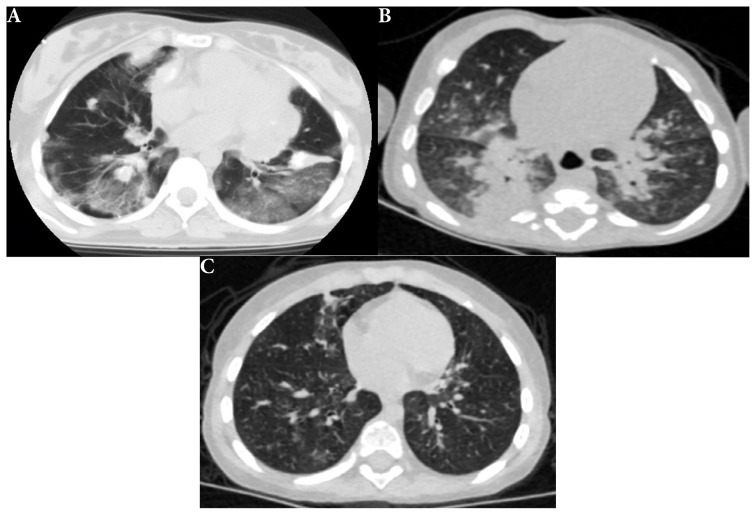
**A** Patient 190: 17-year-old girl, metastatic Ewing’s sarcoma. Lower lobe predominance peripheral ground glass infiltrations and metastases in both lungs and mediastinum. PCR test for COVID-19 was positive, **B)** Patient 40: 1-year-old boy with Schinzel Giedion syndrome (the same patient as in Figure 1B). Patchy consolidations at perihilar regions in both lungs. Rhinovirus was detected in RVP tests, **C**) Patient 27: 3-year-old girl with immune deficiency syndrome (chronic granulomatous disease). Peribronchovascular infiltrations in both lungs. Diagnosis of RSV pneumonia was made after RVP tests.

The CT scans of 11 (25.6%) of the COVID-19-positive and 8 (21.6%) of the RVP positive patients [for a total of 19 (23.8%) patients] were normal from the total of 80 patients evaluated with CT without statistically significant difference (p = 0.880) (Figure 5). Multifocal or diffuse ground-glass infiltration and/or consolidation [30 (37.5%)], bilateral lower lobe predominantly peripheral and/or subpleural ground-glass infiltration and/or consolidation [10 (12.5%)], unilateral peripheral ground-glass infiltration and/or consolidation [6 (7.5%)], effusion [16 (20%)], and lymphadenopathy [14 (17.5%)] were all common in both groups, with no statistically significant differences (p = 1.000, p = 0.097, p = 0.681, p = 0.082, p = 0.074, respectively) (Figure 6). Findings such as halo sign in five (6.3%), crazy paving sign in four (5%), and discrete small nodules (tree-in-bud or centrilobular) were detected in 7 (8.8%) patients. Although these findings were more common in COVID-19-positive patients than in RVP-positive patients, there were no statistically significant differences between the groups (p = 0.058, p = 0.120, p = 0.442, respectively). The CT parameters of the patient groups and the comparisons between the groups are presented in Table 2.

In the evaluation between the COVID-19/RSV/rhinovirus three groups—, unilateral, segmental, or lobar—consolidation was more common in rhinovirus cases than in the others [three (25.0%)] (p = 0.010). There were no statistically significant differences between the three groups in findings such as multifocal or diffuse ground-glass infiltration and/or consolidation without specific distribution and unilateral peripheral or peripheral central ground-glass infiltrates and/or consolidation (p = 0.473, p = 0.980, respectively). It was determined that 78 (36.6%) of the patients who underwent X-rays did not have pneumonia. No evidence of pneumonia was detected in the X-ray radiographs taken in 38 (51.4%) of the COVID-19-positive patients and 40 (28.8%) of the RVP-positive patients. There was a significant difference between the groups (p = 0.020). Chest X-rays taken in more than half of COVID-19 positive patients were normal. In 19 (23.8%) of all patients, 11 (25.6%) of COVID-19-positive patients and 8 (21.6%) of RVP-positive patients, the chest CT was normal and there was no statistical difference (p = 0.880). The CT parameters of the patient groups and the comparisons between the groups are given in Table 2.

## 4. Discussion

In lung involvement, chest X-rays are often the first radiological method preferred due to low radiation exposure. The most common involvement of COVID-19 pneumonia on chest X-rays in children is peribronchial thickening and multifocal ground-glass infiltrates [5,9,10]. In our study, even if the half of the radiographs showed no abnormality, multifocal or diffuse ground-glass infiltration and/or consolidation and unilateral peripheral or peripheral central ground glass infiltration and/or consolidation were the most common findings observed on chest X-rays in initial evaluation of two days. In previous studies, the most common findings on chest X-rays of COVID-19-positive pediatric patients were reported as peribronchial thickening (58%–86%), ground glass infiltrations (19%–50%), and consolidation (18%–35%) [8,11]. The most common findings on chest X-rays of COVID-19-positive pediatric patients were like those in our study. Chest X-ray and CT findings were evaluated blindly by pediatric radiologists. They concluded that the chest X-ray abnormalities were not specific to COVID-19. Patients with COVID-19 had less peribronchial thickening and/or opacity than RVP-positive patients [11,12].

Unlike others (9%–12%) [12], the rate of COVID-19-positive patients with normal chest radiography (51.4%) was higher in a recent study. However, a study by Palabiyik et al. [13] found normal chest radiography in 54% of patients, similar to our findings (51.4%) [11,13]. In this study, multifocal or diffuse ground-glass infiltration and/or consolidation and unilateral peripheral or peripheral central ground glass infiltration and/or consolidation were the most common findings observed on chest X-rays.

The use of CT imaging in the diagnosis of COVID-19 infection in children is limited due to high-dose radiation exposure. While the CT scan is normal in most children 19 (23.8%), all patients who had chest X-ray findings of disease exhibited various abnormalities. Among these findings, the most common were bilateral peripheral ground-glass infiltrates (10, 12.5%) and unilateral peripheral ground-glass infiltrates (6, 7.5%), along with crazy paving patterns (4, 5%), halo signs (5, 6.3%), and inverted halo signs. Children of all ages are susceptible to COVID-19. However, clinical manifestations are less severe than in adults and, probably as a consequence, the radiologic findings are less marked. Imaging should not be considered a screening tool for diagnosis in children. If imaging is deemed necessary, chest radiography is the preferred initial modality. The CT findings of COVID-19 pneumonia are varied, and their specificity is low (25%) [14,15,16]. While patchy ground-glass infiltrations were identified in studies conducted in the early stages of the pandemic, subsequent studies highlighted lower lobe predominance and bilateral and multifocal involvement [17,18].

Unlike a study by Steinberger et al. [19], in our study, the rates of negative CT examinations with normal imaging findings were relatively low, with 11 (25%) in COVID-19-positive patients. This may be related to the fact that all of the patients included in our study needed intensive care clinically and were followed in the PICU. In previous studies, the most common abnormal findings in CT scans of COVID-19-positive pediatric patients were ground-glass infiltration (86%–88%), consolidation (14%–58%), crazy paving pattern (29%), inverted halo sign (29%), and halo sign (29%) [19]. In the study by Steinberger et al., 86% of the patients were reported to have abnormal findings in the peripheral lung areas, while other studies reported that the abnormal findings detected on CT were in the lower lobes (64%–86%) [19,20]. In our study, abnormal CT imaging findings predominantly involved the lower lobes 8 (18.6%), and the peripheral location of the lesions was seen at a lower rate than in previous studies. CT should be reserved for complex cases, suspected complications, or possible differential diagnoses, particularly in children with associated medical conditions. We can further evaluate lesions, such as crazy pavement sign, halo sign, lung cavitation, which are not visible on X-ray but visible on thorax CT. In our study, the most common finding on CT scans with COVID-19 was multifocal or diffuse GGOs without specific distribution. This finding was observed in RVP-positive patients at a similar ratio.

Our Study’s design was based on the International Expert Consensus Statement on Chest Imaging in Pediatric COVID-19 Patients by Foust et al. [8]. The Radiology Cardiothoracic Imaging consensus in 2020 reported some similarities between imaging findings of COVID-19 and other respiratory infections. In this consensus, only bilateral peribronchial thickening was found to be useful in differentiating RVP factor-associated pneumonia from COVID-19 pneumonia. However, this finding was not remarkable in CT examinations and we could not specifically address whether it was related with treatment or not.

### 4.1. Limitations

There are some limitations to this study. Firstly, the reference standard used was solely RT-PCR results. COVID-19 antibody testing could not be performed for all our cases. Additionally, only the initial radiological images of all patients were evaluated, and subsequent images were not assessed. Therefore, changes such as healed or worsened lesions could not be compared over time. Furthermore, patients with positive test results were included in this study even if their radiological imaging was normal. These patients may have had comorbidities or were previously healthy.

## 5. Conclusion

These observations have shown that viruses other than SARS-CoV-2 may also create ground-glass opacities (GGOs), multiple patchy consolidations, and peripheral or central involvement in the lung parenchyma similar to COVID-19. Although X-ray is essential for managing symptomatic respiratory diseases in children, our study results suggest that CT is neither suitable nor appropriate for excluding COVID-19. Therefore, RVP testing should be considered alongside the RT-PCR test. Specific COVID-19 treatment should not be initiated based solely on thoracic CT findings. COVID-19 PCR and RVP tests should be conducted, and supportive treatment should be provided until the diagnosis is confirmed. Chest X-ray and CT findings in COVID-19 are nonspecific and can be seen in any lower airway infection or pneumonia. Therefore, chest radiographs and CT have a limited role in differentiating COVID-19 from other childhood lung infections. Our study found that the specificity of thoracic CT was low, and it should not be used in children unless absolutely necessary due to radiation-related side effects. It is strongly recommended that thoracic CT should not be performed for diagnosis unless there is severe PARDS.

## Figures and Tables

**Table 1:** Demographic and clinical characteristics of patients infected with COVID-19 and other respiratory tract viruses.

		COVID-19/RVP-positive PCR			COVID-19/rhinovirus/RSV			
	Total (n:225)	COVID-19 (n:81)	RVP (n:144)	p-values	COVID-19	Rhinovirus (n:33)	RSV (n:56)	p-values
**Demographic characteristics**								
Age (month)*,(IQR median	24(7–96)	96(17–156)	17(6–48)	**<0.001**	96(17–156)	11(4–36)	8(3–33)	**<0.001**
Male, no. (%)**	130(57.8)	44(54.3)	86(59.7)	0.431	44(54.3)	14 (42.4)	18 (32.1)	0.271
**Severity of the disease**								
PRISM III* score	7(3–12.5)	8(4–14)	6(3–12)	**0.026**	8(4–14)	8(3–11)	4(2–10.5)	**0.006**
PELOD-2*score	2(1–11)	10(4–11)	1(0–10)	**<0.001**	10(4–11)	3.5(1–11)	1(0–2)	**<0.001**
OSI*	6(3.6–12)	3.65(0–8.35)	7.75(5–13)	**0.016**	3.7(0–8.4)	5.8(4.8–0.2)	7(6–8)	0.126
FiO2*	50(40–60)	40(40–60)	50(40–60)	**0.003**	40(40–60)	50(40–60)	50(40–60)	0.113
**Patient contact** **	50(22.2)	34(42.0)	16(11.1)	**<0.001**	34(42.0)	7(21.2)	7(12.5)	**<0.001**
Comorbidity**	131(59)	46(58.2)	85(59.4)	0.860	46(58.2)	17(53.1)	29(51.8)	0.736
**Cough** **	90(40.0)	29(35.8)	61(42.4)	0.335	29(35.8)	11(33.3)	32(57.1)	**0.023**
**Fever** **	106(47.1)	44(54.3)	62(43.1)	0.104	44(54.3)	9(27.3)	25(44.6)	**0.028**
**Shortness of breath** **	172(76)	47(58)	125(86.8)	**<0.001**	47(58)	26(78.8)	53(94.6)	**<0.001**
Low SpO_2_**(<92%)	57(25.8)	17(21.0)	40(27.8)	0.261	17(21.0)	9(27.3)	12(21.4)	0.758
**Crackles** **	139(61.8)	37(45.7)	102(70.8)	**<0.001**	37(45.7)	18(54.5	43(76.8)	**<0.001**
**Rhonchi** **	93(41.3)	20(24.7)	73(50.7)	**<0.001**	20(24.7)	16(48.5)	35(62.5)	**<0.001**
**Lymphopenia** **	98(44.1)	40(50)	58(40.8)	0.130	40(50)	10(31.3)	19(33.9)	**0.017**
**Leukopenia** **	41(18.2)	17(21)	24(16.7)	0.096	17(21)	4 (12.1)	6(10.7)	**0.038**
Anemia**	120(54.5)	44(55)	76(54.3)	0.217	44(55)	15(46.9)	29(53.7)	0.113
Thrombocytopenia**	51(22.9)	20(25.3	31(21.5)	0.633	20(25.3	5(15.2)	7(12.5)	0.140
**Elevated CRP** **	148(68.2)	56(72.7)	92(65.7)	0.363	56(72.7)	14(43.8)	36(66.7)	**0.017**
Elevated Procalcitonin **	52(41.9)	22(40.7)	30(42.9)	0.958	22(40.7)	3(33.3)	11(34.4)	0.536
**Respiratory Failure** **	183(81.3)	50(61.7)	133(92.4)	**<0.001**	50(61.7)	31(93.9)	50(89.3)	**<0.001**
**Circulatory failure** **	61(27.1)	20(24.7)	41(28.5)	0.648	20(24.7)	13(39.4)	6(10.7)	**0.006**

* ; Median (IQR; 25%–75%),

** ; number (%), RVP: respiratory viral panel, RSV: respiratory syncytial virus, OSI: oxygen saturation index, FiO_2_: fraction of inspired oxygen.

**Table 2:** Radiological comparison of lung involvement of COVID-19 and other respiratory tract viruses.

	Total	COVID-19	RVP(Total)	p-values	Rhinovirus	RSV	p-values
**Chest-X-ray**	n:213	74	139		32	55	
Bilateral distribution peripheral and/or subpleural GGOs and/or consolidation	11(5.2)	5(6.8)	6(4.3)	0.520	1(3.1)	1(1.8)	0.276
Unilateral peripheral or periferocentral GGOs and/or consolidation	27(12.7)	12(16.2)	15(10.8)	0.359	4(12.5)	3(5.5)	0.335
**Bilateral peribronchial thickening and/or peripheral opacities**	37(17.4)	7(9.5)	30(21.6)	**0.042**	7(21.9)	13(23.6)	0.054
Multifocal or diffuse GGOs and/or consolidation without specific distribution	46(21.6)	12(16.2)	34(24.5)	0.223	4(12.5)	11(20)	0.793
Unilateral segmental or lobar consolidation	16(7.5)	4(5.4)	12(8.6)	0.563	6(18.8)	5(9.1)	**0.038**
Central unilateral or bilateral GGOs and/or consolidation	22(10.3)	6(8.1)	16(11.5)	0.589	5(15.6)	7(12.7)	0.230
Single round consolidation (round pneumonia+-air bronchogram)	0(0)	0(0)	0(0)	-	0(0)	0(0)	-
Pleural effusion	27(12.7)	9(12.2)	18(12.9)	1.000	5(15.6)	5(9.1)	0.762
Lymphadenopathy	2(0.9)	1(1.4)	1(0.7)	1.000	1(3.1)	0(0)	0.630
**No findings suggestive of pneumonia**	78(36.6)	38(51.4)	40(28.8)	**0.020**	9(28.1)	18(32.7)	**0.029**
CT	n:80	43	37		12	8	
Bilateral peripheral and/or subpleural GGOs and/or consolidation, with a lower lobe predominant pattern	10(12.5)	8(18.6)	2(5.4)	0.097	0(0)	0(0)	0.055
Halo sign	5(6.3)	5(11.6)	0(0)	0.058	0(0)	0(0)	0.139
Unilateral peripheral or periferocentral GGOs and/or consolidation	6(7.5)	4(9.3)	2(5.4)	0.681	1(8.3)	1(12.5)	0.980
Bilateral peribronchial thickening and/or peribronchial opacities	8(10)	3(7)	5(13.5)	0.461	0(0)	1(12.5)	0.506
Multifocal or diffuse GGOs and/or consolidation without specific distribution	30(37.5)	16(37.2)	14(37.8)	1.000	3(25)	3(37.5)	0.473
Crazy paving sign	4(5)	4(9.3)	0(0)	0.120	0(0)	0(0)	0.190
Unilateral segmental or lobar consolidation	5(6.3)	1(2.3)	4(10.8)	0.176	3(25)	0(0)	**0.010**
Central unilateral or bilateral GGOs and/or consolidation	3(3.8)	0(0)	3(8.1)	0.095	1(8.3)	1(12.5)	0.075
Discrete small nodules (tree in bud, centricolbular)	7(8.8)	5(11.6)	2(5.4)	0.442	1(8.3)	0(0)	0.574
Lung cavitation	2(2.5)	1(2.4)	1(2.7)	1.000	0(0)	0(0)	0.508
Pleural effusion	16(20)	5(11.6)	11(29.7)	0.082	2(16.7)	0(0)	0.824
Lymphadenopathy	14(17.5)	4(9.3)	10(27)	0.074	4(33.3)	0(0)	0.063
No findings suggestive of pneumonia	19(23.8)	11(25.6)	8(21.6)	0.880	3(25)	3(37.5)	0.897

X-ray: Chest X-ray, CT: computed tomography, GGOs: ground-glass opacities, RSV: respiratory syncytial virus.
